# In growing lambs, can body weight be predicted from the dorsal area?

**DOI:** 10.1093/tas/txag089

**Published:** 2026-06-29

**Authors:** Miguel Ángel Gastelum-Delgado, Roberto Carlos Barrientos-Medina, Darwin Nicolas Arcos-Álvarez, Ignacio Vázquez-Martínez, Jesus Mezo-Solis, Enrique Camacho Perez, Tairon Pannunzio Dias-Silva, Antonio Leandro Chaves Gurgel, Alfonso Juventino Chay-Canul

**Affiliations:** División Académica de Ciencias Agropecuarias, Carretera Villahermosa-Teapa, km 25, R/A. La Huasteca 2, Universidad Juárez Autónoma de Tabasco, Sección, Centro, Tabasco, México. C.P, Tabasco, Villahermosa 86280, México; Facultad de Medicina Veterinaria y Zootecnia, Campus de Ciencias Biológicas y Agropecuarias, Universidad Autónoma de Yucatán, km 15.5 carretera Mérida-Xmatkuil, Mérida, Yucatán C. P. 97135, México; División de Estudios de Posgrado e Investigación, Tecnológico Nacional de México, Campus Conkal, Avenida Tecnológico S/N, Conkal, Yucatán, México; División Académica de Ciencias Agropecuarias, Carretera Villahermosa-Teapa, km 25, R/A. La Huasteca 2, Universidad Juárez Autónoma de Tabasco, Sección, Centro, Tabasco, México. C.P, Tabasco, Villahermosa 86280, México; División de Estudios de Posgrado e Investigación, Tecnológico Nacional de México, Campus Conkal, Avenida Tecnológico S/N, Conkal, Yucatán, México; Industrias No Contaminantes s/n, Universidad Autónoma de Yucatán, Facultad de Ingeniería. Av, Mérida, Yucatán, CP 97000, México; Campus Professora Cinobelina Elvas, Universidade Federal do Piauí, Bom Jesus, Piauí 64900-000, Brasil; Campus Professora Cinobelina Elvas, Universidade Federal do Piauí, Bom Jesus, Piauí 64900-000, Brasil; División Académica de Ciencias Agropecuarias, Carretera Villahermosa-Teapa, km 25, R/A. La Huasteca 2, Universidad Juárez Autónoma de Tabasco, Sección, Centro, Tabasco, México. C.P, Tabasco, Villahermosa 86280, México

**Keywords:** body weight, biometric measurements, hair sheep, humid tropics, mathematical models

## Abstract

Periodic assessment of body weight is one of the main parameters for evaluating the performance of a herd and the consequent efficiency of the production system. Based on this, the aim of the present study was to develop and evaluate different equation models for predicting body weight (BW) in growing lambs using the dorsal area (DA). The variables BW (27.56 ± 5.86 kg), hip width (HW), thoracic width (TW) and body length (BL) were measured in 275 growing male lambs (Pelibuey × Blackbelly × Katahdin) aged between 6 and 9 months and reared under humid tropical conditions. Dorsal area (cm^2^) was calculated using the mathematical formulae for calculating the area of a trapezium, taking into account HW, TW and BL in the calculation. The prediction models were constructed using linear and non-linear regression. Goodness of model fit was assessed using Akaike information criterion (AIC), Bayesian information criterion (BIC), coefficient of determination (*R*^2^), mean square error (MSE) and root mean square error (RMSE). Internal cross-validation (*k*-folds) was also used to evaluate the developed models. The root mean square error of prediction (RMSEP), *R*^2^ and mean absolute error (MAE) were used to assess the ability of the fitted models to predict the observed values. The model selected for the prediction of BW was: −8.47 + 0.06 × DA −2.29 × DA^2^ as it had the lowest MSE, RMSE and AIC values. Furthermore, for the relationship between BW and DA, this quadratic fit gave the highest *R*^2^ value (0.67). Therefore, with a reasonable level of accuracy, the quadratic model using the dorsal area may be suitable for the prediction of body in growing lambs. Based on results, dorsal area may be a moderate predictor of body weight in growing lambs, contributing to better property management.

## Teaser

A significant, moderate and positive correlation was verified between body weight and dorsal area. Furthermore, the multiple parameters used to assess the quality of the regression models indicated a good fit. Thus. Dorsal area may be a moderate predictor of body weight in growing lambs, contributing to better property management.

## Introduction

The use of crossbred sheep in different agro-ecological regions of Mexico has proven to be an important practice, aiming to exploit heterosis (Vargas-Bello-Pérez et al. 2023). In this scenario, breeds such as Pelibuey, Blackbelly and Katahdin have been used for crossbred purposes. The characteristics of easy care, adaptability to a broad range of environmental factors, Rusticity, non-seasonality of reproduction, prolificacy, lamb production and tolerance to internal parasites explain the predominance of these hair sheep in Mexico (Magaña-Monforte et al. 2013; Chay-Canul et al. 2016).

The determination of productive and reproductive parameters is essential for correct management in an agricultural property that seeks efficiency in the adopted system (Robinson, 2024). However, on small properties the level of facilities does not allow the small producer to obtain all the information on the herd for quick and correct decision-making through data (Shahab et al. 2024).

Traditional morphometric measurements such as body length or thoracic perimeter have commonly been used to estimate body weight in sheep because they reflect skeletal growth and body development. However, isolated linear measurements may not fully represent the multidimensional nature of body conformation. In this context, dorsal area (DA) may provide a more integrative assessment of body size because it combines body length, thoracic width, and hip width into a single two-dimensional measurement. Furthermore, DA-based models showed lower prediction errors and improved goodness-of-fit indicators compared with alternative approaches, suggesting that dorsal measurements may represent a practical and biologically meaningful tool for body weight estimation under production conditions.

Regarding management, measuring body weight (BW) is important for designing nutrition and health programs (Sabbioni et al. 2020). In hair sheep, the use of biometric measurements (MBS) has allowed determining the degree of association of a given MBS with some traits of productive interest, such as estimating body weight (Chay-Canul et al. 2019; Canul-Solís et al. 2020), carcass tissue weight (Bautista-Díaz et al. 2017, 2020), and body mass index (Salazar-Cuytun et al. 2020).

On the other hand, the use of three-dimensional cameras to predict liveweight has been developed in recent decades. In this regard, BW has been predicted in dairy cows by measuring thoracic width (TW), hip width (HW) and body length (BL) using three-dimensional cameras as reported by Martins et al. (2020). Similarly, Shalaldeh et al. (2023) reported that TW, HW and BW were included in models to predict muscle, fat and bone weights in Coopworth sheep using computed tomography to determine body composition.

Recently, Gomez-Vazquez et al. (2024)  evaluated the relationship between BW and DA in water buffalo (*Bubalus bubalis*) and concluded that BW and DA were highly correlated (0.96; *p* < 0.001) and concluded that DA is a good predictor of BW in buffalo. It is important to note that the use of multiple parameters is essential to indicate the best prediction equation, since these parameters converge to the best adjustment of the data based on the observed values. However, to our knowledge, no studies have investigated the relationship between dorsal area and BW in hairy sheep. Therefore, the aim of the present study was to develop and evaluate different equation models for predicting body weight in growing lambs using the dorsal area.

## Materials and methods

### Ethics committee and study location

Animals were treated in accordance with the ethical standards for animal research of the Faculty of Agricultural Sciences of the Autonomous University of Tabasco (CIEI: FOLIO 1173–2022). The experiment was carried out at the Centro de Integración Ovina del Sureste (CIOS, 17°78'N, 92°96'W'; 10 masl). It is in R/a Alvarado Santa Irene 2 da Secc, municipality of Centro, Tabasco, Mexico. It has a humid tropical climate. Temperatures range from 15 to 44°C, with an average of 26°C.

### Data collection

**Figure 1:**
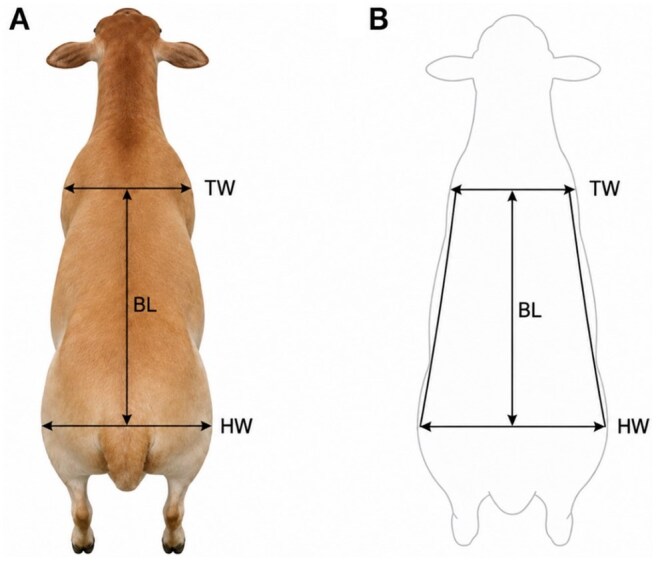
Anatomical landmarks and graphical representation used for dorsal area (DA) determination in hair sheep. Hip width (HW), thoracic width (TW), and body length (BL) were measured to calculate DA using the trapezium area equation.

Data were collected from 275 male hair lambs (Pelibuey × Blackbelly × Katahdin) for Body weight (BW, kg), hip width (HW, cm), thoracic width (TW, cm) and body length (BL, cm). The lambs were clinically healthy and aged between 6 and 9 months. For dorsal area (DA) estimation (Fig. 1), the HW, TW and BL were measured using a flexible fibreglass tape measure (Truper) as described by Bautista-Díaz et al. (2020). Body weight was recorded using a fixed platform scale with a capacity of 600 kg and an accuracy of 20 g, while TW and HW were recorded using a large 65-cm calipers (Haglof).

The DA (cm^2^) was calculated using the mathematical formula for calculating the area of a trapezium as described by Gomez-Vázquez et al. (2024), considering HW, TW and BL in the calculation. The DA was calculated as follows: Dorsal area (cm2) =(thoracic width + hip width)/2 × body length

### Data analysis

For statistical analysis and internal validation of the models, the data were processed in the following way in the Python environment: descriptive statistics were performed using the “describe” function of the “pandas” package (Mckinney, 2010). Linear (Equation 1), quadratic (Equation 2) and allometric (Equation 3) relationships were determined using the “lmfit” package (Newville et al. 2014). The allometric equation fitted was: *Y* = *aX* × *b*, where *Y* is BW, *X* is DA, and *a* and *b* are model parameters. The matplotlib package was used to plot the models and their residuals (Hunter, 2007). The Akaike information criterion (AIC), Bayesian information criterion (BIC), coefficient of determination (*R*^2^), mean square error (MSE) and root mean square error (RMSE) were used to assess the goodness of fit of the regression models. The last three parameters were obtained using the scikit-learn package (Pedregosa et al. 2011).

Cross-validation of *k*-folds (*k* = 4) was used to assess the predictive ability of the three models for BW. In this approach, the set of observations was randomly divided into a number of non-overlapping *k*-folds of approximately the same size. The model was fitted to the remaining *k*-1 folds (training data), with the first fold treated as the validation set. The MSE, *R*^2^ and mean absolute error (MAE) were used to assess the ability of the fitted model to predict the actual observations. The MAE refers to the mean absolute difference between observed and predicted results and is an alternative to the mean squared prediction error (MSPE), which is less sensitive to outliers. Lower values of the root-mean-square (RMSPE) and MAE are an indication of a better fit. The *k*-fold cross-validation was performed using the “scikit-learn” package. This allows the comparison of a large number of multivariate calibration models.

## Results

The growing lambs presented BW and DA of 27.56 ± 5.86 kg and 840.58 ± 198.43 cm^2^, respectively (Table 1). Also, a positive, moderate and significant, (0.79, *p* < 0.0001) correlation was verified between BW and DA.

**Table 1:** Minimum and maximum values for body weight (BW) and dorsal area (DA) in hair sheep (*n* = 275) kept under humid tropical conditions.

Variables	*N*	Mean ± *SD*	Minimum	Maximum	CV (%)
**Body weight (kg)**	275	27.56 ± 5.86	11.00	42.90	15.43
**Dorsal area (cm^2^)**	275	840.58 ± 198.43	405	1513.00	38.21

*SD*, standard deviation.

Linear, quadratic, and allometric equations were significant (*p* < 0.05) for estimating BW based on DA. Among the evaluated models, the parameters R^2^, MSE, RMSE, AIC, and BIC indicated that the quadratic regression equation provided the best fit for estimating body weight from dorsal area when compared with the linear and allometric models (Table 2).

**Table 2:** Regression equations to estimate body weight (BW) and dorsal area (DA) in hair sheep kept under humid tropical conditions.

No	Equation	*R^2^*	MSE	RMSE	AIC	BIC	*P*-value
**1**	BW (kg): 7.89 (±0.94***) + 0.02 (±0.0019***) × DA	0.62	12.80	3.58	705.00	712.24	<0.0001
**2**	BW (kg): −8.47 (±0.0001***) + 0.06 (±0.006***) × DA −2.29 (±0.0001*) × DA^2^	0.67	11.26	3.35	671.72	682.57	<0.0001
**3**	BW (kg): 0.25 (±0.06***) × DA^0.69 (±0.03*)^	0.64	12.27	3.50	693.47	700.70	<0.0001

BW, body weight; *R^2^*, Coefficient of determination; MSE, mean square error; RMSE, Root MSE; AIC, Akaike Information Criterion; BIC, Bayesian Information Criterion. Values in parentheses are the parameter estimates’ standard errors (*SE*); The * indicates:

* *p* < 0.05;

** *p* < 0.01;

*** *p* < 0.001; Equation 1: Linear; Equation 2: Quadratic; Equation 3: Allometric.

The superiority of the quadratic model was further confirmed through k-fold cross-validation. Evaluation and validation indicators, including *R*^2^, MSPE, and MAE, demonstrated that the quadratic regression model showed greater predictive accuracy and robustness than the other tested equations (Table 3).

**Table 3:** Internal *k*-folds cross-validation of the proposed models.

Model	*N*	*R^2^*	MSPE	MAE
**Linear**	275	0.36	3.59	3.07
**Quadratic**	275	0.47	3.38	2.82
**Allometric**	275	0.41	3.52	3.00

*N*, number of observations; MSPE, mean squared prediction error; *R^2^*, coefficient of determination; MAE, mean absolute error.

The quadratic model provided the best fit for predicting BW from DA, indicating that the relationship between both variables was non-linear. The fitted curve showed that BW increased progressively with increasing DA, although the rate of increase was not constant across the evaluated range. At lower DA values, BW increased more rapidly, whereas at larger DA values the curve tended to stabilise, suggesting a reduction in the proportional increase in BW relative to dorsal expansion. This behaviour may reflect changes in growth dynamics and body tissue deposition as animals advance in age and body development.

In addition, the quadratic model presented a mean absolute error (MAE) of 2.82 kg, indicating that predicted BW values differed on average by approximately ± 2.8 kg from observed BW under the evaluated conditions. From a practical perspective, this result demonstrates satisfactory predictive precision and provides an estimate of the expected prediction error when the equation is applied under field conditions.

## Discussion

Based on the present study, which includes multiple parameters to assess the goodness of fit of the regression models (*R*^2^, MSE, RMSE, AIC e BIC), it can be stated that the body weight of growing lambs can be predicted using the calculation of the dorsal area of these animals. In this context, this study represents the first use of DA as an instrument for estimating body weight in growing lambs, Further study may be necessary, including new tools to increase the response power of the models in explaining the variable analyzed.

It has been noted in the scientific literature that different models have been developed for the estimation of BW from body measurements such as heart girth (HG) in different breeds of small ruminants (Atta et al. 2024). In this context, Atta et al. (2024) reported that HG was the best linear measure for predicting BW in goats. The HG has been shown to be the biometric measure with the highest correlation with BW in adult hair sheep in previous studies (Chay-Canul et al. 2019; Canul-Solís et al. 2020). HG has certain advantages over other BMs, such as the ease with which it can be measured during routine management practices, as it does not require any special facilities and requires less handling of the animal (Chay-Canul et al. 2019). In this context, the present study used data from HW, TW and BL to calculate the dorsal area of animals, which can effectively be used as a tool to estimate the body weight of animals, helping in decision-making within the production system adopted on farms. However, it should be noted that the DA showed moderate effectiveness in predicting BW of growing lambs. The moderate validation performance observed for the quadratic model (*R*^2^ = 0.47) may be partially explained by the biological heterogeneity of the evaluated animals. Although dorsal area integrates body length, thoracic width, and hip width, the relationship between body surface and body weight can vary according to age, breed type, and body conformation. In the present study, lambs ranged from 3 to 9 months of age, a period characterized by rapid changes in skeletal growth, muscle accretion, and fat deposition. Therefore, animals with similar dorsal areas may differ in body density and tissue composition, reducing the strength of the observed-predicted relationship during validation.

Additionally, the inclusion of different hair sheep genotypes, such as Pelibuey, Blackbelly, and Katahdin, may have contributed to the lower validation coefficient. These breeds differ in mature size, growth pattern, body shape, and tissue deposition potential. Consequently, a single equation based on dorsal area may not fully capture breed-specific differences in body conformation. This suggests that future studies should evaluate genotype-specific or breed-adjusted models to improve prediction accuracy.

In addition, image processing technologies have developed rapidly in recent decades, and digital image analysis (DIA) is currently used in many scientific fields, including veterinary and animal production sciences (Chay-Canul et al. 2023). In this sense, the determination of BW in livestock by image analysis is an emerging area of research, offering the possibility of automatically measuring the dimensions of animal images and using BW prediction equations (Chay-Canul et al. 2023). BW prediction is based on the use of lateral and dorsal images of the animal. These views provide different BMs such as withers height, rump height, body length, diagonal body length, ridge depth, chest depth, chest width, thoracic width, abdominal width and dorsal height, which can be correlated with BW (Chay-Canul et al. 2023).

**Figure 2:**
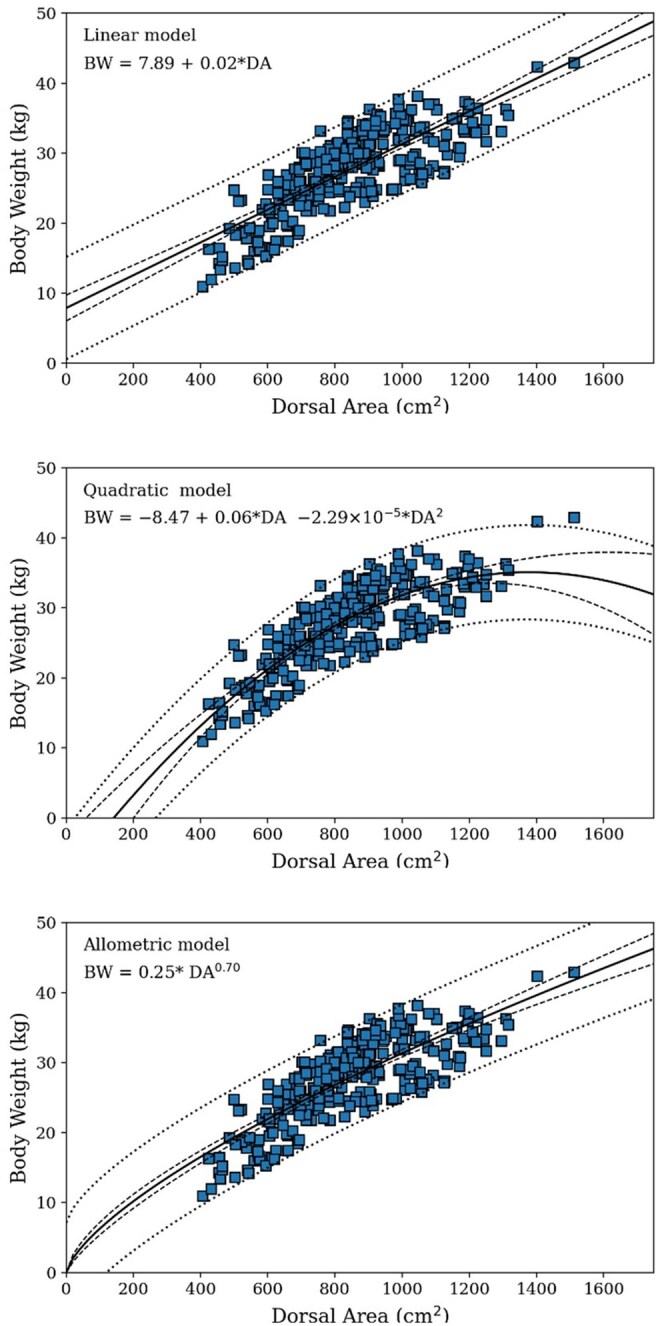
Scatterplot of dorsal area and body weight of animals: (1) linear model, (2) quadratic model and (3) allometric model; BW—body weight; DA—dorsal area.

In this study we evaluated the use of DA to estimate BW in growing lambs and found that, via internal k-fold cross-validation, the quadratic model showed a better fit to the data (Table 3 and Fig. 2), with significant contributions in both the quadratic and allometric terms (*p* < 0.0001 in both cases). The superior fit of the quadratic model suggests that the relationship between dorsal area and body weight is not strictly proportional during growth. From an allometric perspective, body development in growing lambs involves progressive changes in tissue composition and body conformation rather than uniform expansion of body dimensions. As animals advance from approximately 3 to 9 months of age, muscle accretion, skeletal growth, and fat deposition occur at different rates, modifying body density and shape over time. Consequently, increases in dorsal area are not accompanied by constant increases in body weight across all developmental stages.

At earlier growth stages, increases in dorsal area may be associated primarily with skeletal and muscular development, whereas at later stages tissue deposition, particularly fat accumulation, may contribute disproportionately to body weight relative to surface expansion. Therefore, the quadratic model may better capture these non-linear biological changes in body composition and growth dynamics than a simple linear approach.

Recently, Gómez-Vázquez et al. (2024) evaluated equations for predicting BW in buffalo using DA as a predictor and reported that the quadratic equation had the lower values of mean square error of prediction and mean absolute error when the fit was evaluated using the k-fold technique. These authors concluded that DA can be a good predictor of BW in buffalo, especially when included in first and second order linear equations. They also concluded that their results suggest that in future studies, digital photography of the dorsal view of animals could be used to calculate DA using biometric measurements and obtain a good relationship with BW. It is worth noting that the study by Gómez-Vázquez et al. (2024) represents an innovative use of the DA as a tool for the estimation of the body weight of buffalo.

Chest circumference is traditionally considered the main morphometric measurement for predicting body weight in small ruminants due to its high correlation with animal mass. However, dorsal area obtained through image analysis represents a promising alternative because it enables non-invasive measurements with reduced animal restraint and lower operator-dependent variability. In addition, image-based methods may facilitate automation and real-time monitoring under field conditions. Although the present study did not directly compare dorsal area and chest circumference under the same experimental conditions, the predictive accuracy obtained for dorsal area suggests that this approach has potential as a complementary or alternative tool for estimating body weight in sheep.

## Conclusions

Dorsal area, based on hip width, thoracic width and body length, may be a moderate predictor of body weight in growing lambs. However, these results suggest that there is a need for further studies that include digital photography of the dorsal view of the animals to calculate the dorsal area using biometric measurements and to evaluate the relationship with body weight in different sheep categories.

## Data Availability

The data and developed models will be deposited upon reasonable request.

## List of abbreviations

BW: body weight
DA: dorsal area
HW: hip width
TW: thoracic width
BL: body length
AIC: Akaike information criterion
BIC: Bayesian information criterion
*R* ^2^: coefficient of determination
MSE: mean square error
RMSE: root mean square error
